# ERCC1、RRM1和TS在非小细胞肺癌中的临床意义

**DOI:** 10.3779/j.issn.1009-3419.2014.06.11

**Published:** 2014-06-20

**Authors:** 培 李, 晓征 康, 克能 陈

**Affiliations:** 100142 北京，北京大学肿瘤医院暨恶性肿瘤发病机制及转化研究教育部重点实验室胸外一科 Key laboratory of Carcinogenesis and Translational Research (Ministry of Education), Department I of Thoracic Surgery, Peking University Cancer Hospital, Beijing 100142, China

肺癌为临床常见恶性肿瘤，2014年肺癌约占所有恶性肿瘤死亡率的1/4，约占所有新发肿瘤的14%^[1]^，其中非小细胞肺癌（non-small cell lung cancer, NSCLC）约80%^[2]^。目前临床对NSCLC广泛采纳以解剖为基础的TNM（tumor-node-metastasis）分期及组织病理学作为治疗及预后判断的标准，但近年来出现许多指导NSCLC治疗策略的新观念，典型例子是表皮生长因子受体（epidermal growth factor receptor, *EGFR*）突变与EGFR-酪氨酸激酶抑制剂（tyrosine-kinase inhibitor, TKI）治疗，*ALK*基因重排与克唑替尼治疗的故事等。此外生物标志物对肺癌的经典治疗——化疗疗效的预测研究也不断被报道，如目前认为核苷酸切除修复交叉互补组（excision repair cross-complementing 1, ERCC1）、核苷酸还原酶M1（ribonucleotide reductase subunit 1, RRM1）、胸苷酸合成酶（thymidylate synthase, TS）表达水平与NSCLC治疗与预后密切相关，其中ERCC1与肺癌铂类治疗耐药及预后相关，RRM1与吉西他滨治疗耐药及预后相关，TS与培美曲塞耐药及预后相关。2009年美国国立综合癌症网络（National Comprehensive Cancer Network, NCCN）推荐ERCC1和RRM1蛋白表达与*EGFR*基因突变一起，作为评估肺癌化疗后预后与预测治疗的标记物。本文就ERCC1、RRM1及TS在NSCLC中的意义尤其是生存意义做一简要综述。

## 1 ERCC1

### 1.1 概况

ERCC1是体内重要的DNA修复酶的编码基因，其编码的蛋白是核酸切除修复途径（nucleotide excision repair, NER）的限速酶，而NER是DNA切除修复的主要途径^[3]^，*ERCC1*基因位于染色体19q13.2-q13.3，基因全长15 kb，含10个外显子，编码297个氨基酸的蛋白，与着色性干皮病基因F（xeroderma pigmentosum pomp lementary group F, XPF）形成异二聚体ERCC1-XPF，在DNA单链受损处的5’端进行识别和剪切而发挥功能^[4]^，因此，ERCC1在肿瘤的发生发展及预后中发挥着巨大的效用。临床常用免疫组织化学（immunohistochemistry, IHC）或实时定量聚合酶链式反应（quantitative real-time polymerase chain reaction, qRT-PCR）检测ERCC1表达，IHC较qRT-PCR使用率高，一方面由于IHC方法简易、性价比高；另一方面IHC更能直接显示肿瘤细胞中ERCC1蛋白表达的百分数和强度，而qRT-PCR仅显示肿瘤及周边非肿瘤组织混合表达的整体情况。IHC检测ERCC1表达的蛋白的水平，qRT-PCR检测mRNA水平。IHC检测ERCC1蛋白阳性者镜下显示细胞核中出现棕黄色颗粒。

### 1.2 ERCC1作用机制

ERCC1是NER的限速酶，负责对DNA的损伤进行识别和剪切，避免复制错误。而NER是DNA切除修复的主要途径^[3]^，修复机制是通过切除吸烟、大气污染、电离辐射等理化因素损伤的DNA，而形成的DNA加合物，以互补链为模板复制并修复损伤DNA来维护基因组的完整。因此，ERCC1通过控制NER而最终控制DNA的复制。

### 1.3 ERCC1对NSCLC预后及疗效的预测

#### 1.3.1 ERCC1在含铂方案中的作用

近年来研究^[5]^报道ERCC1的表达与肺癌预后及铂类化疗耐药有关。铂类作为NSCLC联合化疗应用最广泛的药物，以铂类药物为基础的双药联合方案目前仍然是NSCLC尤其是晚期NSCLC的一线化疗方案，但晚期NSCLC有效率仅为30%-40%，中位生存期8个月-12个月^[6]^。顺铂引起的损伤主要由细胞内NER进行切除修复^[7]^，其作用机制是ERCC1与肿瘤细胞的DNA进行交联致DNA的损伤及断裂，从而抑制肿瘤细胞的不断增殖。

#### 1.3.2 ERCC1在可手术切除的NSCLC治疗与预后

Simon等^[8]^对51例可手术切除的NSCLC术后组织中ERCC1 mRNA水平采用反转录聚合酶链式反应（reverse transcription-PCR, RT-PCR）检测，发现ERCC1表达水平与患者预后呈正相关，即高表达组的中位生存期明显优于低表达组，中位生存期分别为94.6个月与34.5个月（*P*=0.01）。为明确ERCC1能否预测铂类联合方案在NSCLC术后辅助化疗的疗效，Olaussen等^[9]^采用IHC方法检测国际肺癌临床试验（International Adjuvant Lung Trial, IALT）入组的761例NSCLC患者手术切除肿瘤组织中的ERCC1的表达情况。发现ERCC1阴性NSCLC患者能从铂类联合辅助化疗中受益（HR=0.65; 95%CI: 0.50-0.86;*P*=0.002），而ERCC1阳性却不能获益（HR=1.14; 95%CI: 0.84-1.55; *P*=0.4）。对于单纯手术的患者，ERCC1阴性患者的预后差。

#### 1.3.3 ERCC1在晚期NSCLC治疗与预后

Lord等^[10]^通过RT-PCR技术回顾性分析了56例晚期（Ⅲb期/Ⅳ期）NSCLC患者ERCC1 mRNA的表达与化疗疗效（吉西他滨联合顺铂）关系，表明ERCC1的表达程度与化疗疗效明显相关，晚期NSCLC肿瘤中的ERCC1 mRNA低表达组中位生存期优于高表达组，分别为61.6周*vs* 20.4周（*P*=0.009）。Cobo等^[11]^根据ERCC1 mRNA表达水平进行了一项选择性应用铂类药物的多中心、随机Ⅲ期对照试验，共入组444例的Ⅳ期NSCLC患者，该研究显示依据ERCC1 mRNA表达水平，对铂类进行选择性用药组优于非选择含铂双药联合方案的传统治疗模式，客观缓解率分别为59.7%与39.3%（*P*=0.02）。高志强、韩宝惠等^[12]^为验证ERCC1表达水平与铂类药物治疗和预后关系，入组222例晚期（Ⅲb期/Ⅳ期）NSCLC患者。采用IHC方法检测ERCC1蛋白在肺癌组织的表达。按2:1的比例随机分为个体化治疗组（*n*=147）及标准治疗组（*n*=75）。标准治疗组采用含铂化疗方案。个体化治疗组中ERCC1蛋白高表达的患者采用不含铂类药物的化疗方案，ERCC1蛋白低表达的患者采用含铂化疗方案。结果显示个体化治疗组的中位生存期优于对照组（13.3个月*vs* 10.2个月，*P*=0.041）。

## 2 RRM1

### 2.1 RRM1概况

*RRM1*基因通过编码合成核糖核苷酸还原酶的亚基M1^[13]^。其编码的蛋白RRM1是DNA合成通路中的限速酶，在DNA合成中起到限速、调节作用，*RRM1*基因位于染色体11p15.5，包含19个外显子，编码792氨基酸蛋白质^[14]^。临床工作中常用IHC和qRT-PCR检测RRM1，蛋白表达水平的检测常用IHC技术，IHC检测RRM1蛋白阳性染色定位于细胞质，镜下显示为胞质中出现的大小不一的棕黄色染色颗粒。近期研究^[15]^显示，使用精确定量分析（accurate quantitative analysis, AQUA）检测RRM1蛋白评分方法明显优于IHC的H-评分，因此，AQUA检测RRM1蛋白的方法在未来将可能会广泛地取代IHC和qRT-PCR方法。

### 2.2 RRM1作用机制

RRM1是核苷酸结合位点之一，它与RRM2共同构成核糖核苷酸还原酶（ribonucleotide reductase, RR），RR是DNA合成的限速酶，该酶作用是使二磷酸核苷酸（ribonucleoside diphosphates, rNDP）转化为二磷酸脱氧核苷酸（deoxyribonucleoside diphosphates, dNDP），dNDP是DNA合成和修复必需原料的前体，因此，RRM1通过控制DNA的合成从而控制细胞的增殖。

### 2.3 RRM1预后及疗效预测

#### 2.3.1 RRM1在含吉西他滨化疗方案中的运用

吉西他滨是嘧啶类抗代谢药物，吉西他滨进入细胞内后经过核苷激酶的作用转化成具有活性的二磷酸核苷（5-diphosphate, dFdCDP）及三磷酸核苷（5-triphosphate, dFdCTP），通过抑制DNA合成而发挥细胞毒作用^[16]^，首先，dFdCDP抑制RR的活性，使合成DNA必需的三磷酸脱氧核苷产生减少，尤其使dCTP（deoxycytidine 5-triphosphate）产生减少，dCTP是DNA合成的原料，使DNA合成减少。其次，dFdCTP与dCTP竞争掺入至DNA链中，DNA聚合酶不能去除掺入的吉西他滨及修复延长的DNA链，从而引起DNA链断裂最终导致细胞凋亡。

#### 2.3.2 RRM1在可手术切除的NSCLC治疗与预后

Bepler等^[17]^应用qRT-PCR方法对77例早期NSCLC术后标本中RRM1表达情况进行了前瞻性研究，结果提示RRM1是决定预后的独立危险因素，显示在多变量分析中，RRM1独立于肿瘤的分期、一般状况、体重丢失等，可作为手术切除的NSCLC术后独立的预后因子（HR=0.452, 95%CI: 0.203-1.006），其高表达组较低表达组拥有更长的生存，更低的复发率。Zheng等^[5]^报道187例未接受任何辅助化疗患者，通过根治性切除肿瘤标本进行RRM1蛋白水平的预后价值研究，显示RRM1蛋白高表达组总生存时间优于RRM1蛋白低表达组（120个月*vs* 60.2个月；*P*=0.02）。

#### 2.3.3 RRM1在晚期NSCLC治疗与预后

晚期NSCLC患者中，RRM1的预后价值，主要通过以吉西他滨为基础联合铂类治疗方案来进行研究^[18]^，Rosell等^[19]^通过一组吉西他滨联合顺铂治疗的患者的RRM1 mRNA水平对预后生存的潜在预测价值分析，显示RRM1 mRNA低表达水平组中位生存期明显优于RRM1 mRNA高表达组（13.7个月*vs* 3.6个月，*P*=0.009）。Lee等^[20]^通过IHC检测40例采用吉西他滨治疗的晚期NSCLC患者的RRM1蛋白表达水平，显示RRMl阳性患者在生存期（5.1个月*vs* 12.9个月，*P*=0.022）和疾病控制率（23% *vs* 56%, *P*=0.053）方面均较RRM1阴性差。Rosell和Lee的研究中，均显示晚期的NSCLC中RRM1表达与疾病的预后呈负相关。

## 3 TS

### 3.1 TS概况

TS是一种叶酸依赖性酶，由两个相同的亚基构成，是DNA合成的关键酶，也是培美曲塞、5-氟尿嘧啶（5-fluorouracil, 5-FU）为基础化疗的靶向酶^[21]^。TS是培美曲塞等叶酸抑制剂的的重要作用靶点，其基因定位于人18号染色体短臂18p11.32，长约16×10^3^bp^[22]^。临床中常用IHC和qRT-PCR两种方法检测TS，qRT-PCR检测TS敏感性高，但需要足量的新鲜肿瘤组织标本，且检测费用昂贵。IHC检测TS更常用，因为方法相对简单，此外，既往研究中的IHC采用H-评分法可以得到一致的结果，IHC检测TS表达阳性的肿瘤细胞的细胞质被染成棕褐色。

### 3.2 TS作用机制

TS是体内调节四种核苷酸数量平衡的关键酶，TS通过甲基化脱氧尿嘧啶核苷酸（deoxyuridine monophosphate, dUMP）形成脱氧胸嘧啶核苷酸（deoxythymidine monophosphate, dTMP），dTMP进一步在细胞内代谢为三磷酸胸嘧啶，三磷酸胸嘧啶为DNA合成和修复的基本原料，因此，TS是dTMP从头合成的限速酶，在DNA合成与修复、细胞增殖与分化中起非常重要的作用^[23]^。并且TS是叶酸代谢关键酶，叶酸是核苷酸和DNA合成及甲基化的重要前体物质。低叶酸水平是引起DNA链断裂，导致遗传物质不稳定和癌症风险增加的关键决定因素^[24]^。

### 3.3 TS预后及疗效预测

#### 3.3.1 TS在含培美曲塞化疗方案中的作用

NCCN指南已将培美曲塞（pemetrexed）推荐为晚期NSCLC中腺癌的标准治疗方案之一。TS参与DNA复制和修复，是主要的叶酸依赖酶，培美曲塞作为TS的新一代的多靶点抗叶酸制剂，它在体内产生的多聚谷胺酸代谢物来抑制包括TS、二氢叶酸还原酶（dihydrofolate reductase）和甘氨酰胺核苷甲酰转移酶（glycinamide ribonucleotide formyltransferase），而被抑制的这几种酶是参与嘌呤和嘧啶合成的关键酶^[25]^。根据Liu等^[26]^纳入8项研究的荟萃分析显示TS低表达的患者可从培美曲塞化疗中明显获益（PFS: HR=0.63, 95%CI: 0.52-0.76; OS: HR=0.74, 95%CI: 0.63-0.88），TS表达水平的增加可能是培美曲塞耐药原因。

#### 3.3.2 TS与组织学的关系及预后

Nakagawa等^[27]^通过IHC测定109例Ⅰ期根治性手术切除NSCLC肿瘤组织中TS活性，研究其与肿瘤增殖程度的关系，结果显示肿瘤组织中TS高表达组肿瘤增殖率明显高于TS低表达组，分别为48.2%和34.4%。提示肿瘤组织TS水平越高，癌细胞的增殖活性越高（*P*=0.020）。郭惠琴等^[28]^通过IHC检测111例NSCLC患者的肿瘤标本TS表达情况，表明鳞癌患者的肿瘤组织中TS表达水平较其他组织学类型高（*P*=0.031）。既往Ⅲ期临床试验^[29]^证实，培美曲塞治疗肺腺癌的疗效好于肺鳞癌。TS在鳞癌患者的肿瘤组织中表达水平较其他组织学类型高。Ceppi等^[30]^在56例NSCLC标本中发现TS mRNA水平明显高于腺癌（2.17*vs* 1.16, *P* < 0.000, 1）。Peterson等^[31]^对培美曲赛和多西他赛二线治疗NSCLC头对头的Ⅲ期随机研究进行的回顾性分析发现，在对鳞癌的患者中多西他赛疗效好于培美曲赛；在对非鳞癌的患者，培美曲赛疗效好于多西他赛。同样的Sun等^[32]^通过IHC检测使用培美曲塞为基础的化疗方案的285例非鳞癌的NSCLC的TS蛋白，在多变量分析中，以培美曲塞为基础的化疗TS阴性组对比阳性组有更长的无进展生存期（HR=0.7, 95%CI: 0.51-0.97）。

## 4 小结

综上所述，ERCC1、RRM1及TS均是保证DNA不断复制的关键酶，虽然目前的研究显示的数据表明生物标记物表达水平不同，将直接影响到相关药物的疗效，根据基因表达的效果而选择对应的药物，从而达到更佳的治疗目的，但是，大多数均为回顾性的研究，少数为前瞻性研究。就目前的研究结果并不能作为临床常规诊疗的标准，因此大家更广泛的接受以病理类型、临床分期决定患者的治疗方案。化疗又是NSCLC的主要治疗方式之一，特别是晚期NSCLC患者，不同的个体对化疗的敏感性存在较大差异，这种差异使得医生在面对不同的患者而选择不同的方法，个体化的治疗越来越凸显，因此，针对分子标志物表达的不同及耐药的关系而选择个体化治疗，从而避免过度治疗或治疗不够，将仍然是NSCLC研究的重要内容之一。

## References

[1] Siegel R, Ma J, Zou Z (2014). Cancer statistics, 2014. CA Cancer J Clin.

[2] D'Addario G, Felip E (2009). Non-small-cell lung cancer: ESMO clinical recommendations for diagnosis, treatment and follow-up. Ann Oncol.

[3] Altaha R, Liang X, Yu JJ (2004). Excision repair cross complementing-group 1: Gene expression and platinum resistance. Int J Mol Med.

[4] McVey M, Lee SE (2008). MMEJ repair of double-strand breaks (director's cut): deleted sequences and alternative endings. Trends Genet.

[5] Zheng Z, Chen T, Li X (2007). DNA synthesis and repair genes RRM1 and ERCC1 in lung cancer. N Engl J Med.

[6] Goffin J, Lacchetti C, Ellis PM (2010). First-line systemic chemotherapy in the treatment of advanced non-small cell lung cancer: A systematic review. J Thorac Oncol.

[7] Rosell R, Lord RV, Taron M (2002). DNA repair and cisplatin resistance in non-small-cell lung cancer. Lung Cancer.

[8] Simon GR, Sharma S, Cantor A (2005). ERCC1 expression is a predictor of survival in resected patients with non-small cell lung cancer. Chest.

[9] Olaussen KA, Dunant A, Fouret P (2006). DNA repair by ERCC1 in non-small-cell lung cancer and cisplatin-based adjuvant chemotherapy. N Engl J Med.

[10] Lord RV, Brabender J, Gandara D (2002). Low ERCC1 expression correlates with prolonged survival after cisplatin plus gemcitabine chemotherapy in non-small cell lung cancer. Clin Cancer Res.

[11] Cobo M, Isla D, Massuti B (2007). Customizing cisplatin based on quantitative excision repair cross-complementing 1 mRNA expression: A phase Ⅲ trial in non-small-cell lung cancer. J Clin Oncol.

[12] Gao ZQ, Han BH, Shen C (2013). Clinical research of individualized therapy in advanced non-small cell lung cancer guiding by detection of ERCC1 protein. Zhongguo Ai Zheng Za Zhi.

[13] Smith BD, Karp JE (2003). Ribonucleotide reductase: An old target with new potential. Leuk Res.

[14] Pitterle DM, Kim YC, Jolicoeur EM (1999). Lung cancer and the human gene for ribonucleotide reductase subunit M1 (RRM1). Mamm Genome.

[15] Bepler G, Olaussen KA, Vataire AL (2011). ERCC1 and RRM1 in the international adjuvant lung trial by automated quantitative *in situ* analysis. Am J Pathol.

[16] Pereira S, Fernandes PA, Ramos MJ (2004). Mechanism for ribonucleotide reductase inactivation by the anticancer drug gemcitabine. J Comput Chem.

[17] Bepler G, Sharma S, Cantor A (2004). RRM1 and PTEN as prognostic parameters for overall and disease-free survival in patients with non-small-cell lung cancer. J Clin Oncol.

[18] Jordheim LP, Seve P, Tredan O (2011). The ribonucleotide reductase large subunit (RRM1) as a predictive factor in patients with cancer. Lancet Oncol.

[19] Rosell R, Danenberg KD, Alberola V (2004). Ribonucleotide reductase messenger RNA expression and survival in gemcitabine/cisplatin-treated advanced non-small cell lung cancer patients. Clin Cancer Res.

[20] Lee JJ, Maeng CH, Baek SK (2010). The immunohistochemical overexpression of ribonucleotide reductase regulatory subunit M1 (RRM1) protein is a predictor of shorter survival to gemcitabine-based chemotherapy in advanced non-small cell lung cancer (NSCLC). Lung Cancer.

[21] Peters GJ, van der Wilt CL, van Triest B (1995). Thymidylate synthase and drug resistance. Eur J Cancer.

[22] Hori T, Takahashi E, Ayusawa D (1990). Regional assignment of the human thymidylate synthase (TS) gene to chromosome band 18p11.32 by nonisotopic *in situ* hybridization. Hum Genet,.

[23] Danenberg PV (1977). Thymidylate synthetase - a target enzyme in cancer chemotherapy. Biochim Biophys Acta.

[24] Koehn EM, Perissinotti LL, Moghram S (2012). Folate binding site of flavin-dependent thymidylate synthase. Proc Natl Acad Sci USA.

[25] Sorensen JB (2011). Pharmacokinetic evaluation of pemetrexed. Expert Opin Drug Metab Toxicol.

[26] Liu Y, Yin TJ, Zhou R (2013). Expression of thymidylate synthase predicts clinical outcomes of pemetrexed-containing chemotherapy for non-small-cell lung cancer: a systemic review and meta-analysis. Cancer Chemother Pharmacol.

[27] Nakagawa T, Tanaka F, Otake Y (2002). Prognostic value of thymidylate synthase expression in patients with p-stage Ⅰ adenocarcinoma of the lung. Lung Cancer.

[28] Guo HQ, Zhao Y, Lu JY (2011). Clinicopathologic feature of thymidylate synthase expression in non-small-cell lung carcinoma. Ai Zheng Jin Zhan.

[29] Scagliotti GV, Parikh P, von PJ (2008). Phase Ⅲ study comparing cisplatin plus gemcitabine with cisplatin plus pemetrexed in chemotherapy-naive patients with advanced-stage non-small-cell lung cancer. J Clin Oncol.

[30] Ceppi P, Volante M, Saviozzi S (2006). Squamous cell carcinoma of the lung compared with other histotypes shows higher messenger RNA and protein levels for thymidylate synthase. Cancer.

[31] Peterson P, Park K, Fossella F (2007). Is pemetrexed more effective in adenocarcinoma and large cell lung cancer than in squamous cell carcinoma? A retrospective analysis of a phase Ⅲ trial of pemetrexed vs docetaxel in previously treated patients with advanced non-small cell lung cancer (NSCLC): P2-328. J Thorac Oncol.

[32] Sun JM, Han J, Ahn JS (2011). Significance of thymidylate synthase and thyroid transcription factor 1 expression in patients with nonsquamous non-small cell lung cancer treated with pemetrexed-based chemotherapy. J Thorac Oncol.

